# Efficacy of an insecticide paint against malaria vectors and nuisance in West Africa - Part 2: Field evaluation

**DOI:** 10.1186/1475-2875-9-341

**Published:** 2010-11-25

**Authors:** Beatriz Mosqueira, Joseph Chabi, Fabrice Chandre, Martin Akogbeto, Jean-Marc Hougard, Pierre Carnevale, Santiago Mas-Coma

**Affiliations:** 1 Departamento de Parasitologa, Facultad de Farmacia, Universidad de Valencia, Av. Vicent Andrés Estellés s/n, 46100 Burjassot, Valencia, Spain; 2 Centre de Recherches Entomologique de Cotonou, 06 BP 2604 Cotonou, Benin; 3 UR016, Institut de Recherche pour le Développement (IRD), BP 64501, 34394 Montpellier Cedex 5, France

## Abstract

**Background:**

Widespread resistance of the main malaria vector *Anopheles gambiae *to pyrethroids reported in many African countries and operational drawbacks to current IRS methods suggest the convenience of exploring new products and approaches for vector control. Insecticide paint Inesfly 5A IGR™, containing two organophosphates (OPs), chlorpyrifos and diazinon, and one insect growth regulator (IGR), pyriproxyfen, was tested in Benin, West Africa, for 12 months.

**Methods:**

Field trials were conducted in six experimental huts that were randomly allocated to one or two layers of insecticide at 1 Kg/6 m^2 ^or control. Evaluations included: (i) early mosquito collection, (ii) mosquito release experiments, (iii) residual efficacy tests and (iv) distance tests. Early mosquito collections were performed on local populations of pyrethroid-resistant *An. gambiae *and *Culex quinquefasciatus*. As per WHOPES phase II procedures, four entomological criteria were evaluated: deterrence, excito-repellence, blood-feeding inhibition and mortality. Mosquito release experiments were done using local malaria-free *An. gambiae *females reared at the CREC insectarium. Residual efficacy tests and distance tests were performed using reference susceptible strains of *An. gambiae *and *Cx. quinquefasciatus*.

**Results:**

Six months after treatment, mortality rates were still 90-100% against pyrethroid-resistant mosquito populations in experimental huts. At nine months, mortality rates in huts treated with two layers was still about 90-93% against *An. gambiae *and 55% against *Cx. quinquefasciatus*. Malaria-free local mosquito release experiments yielded a 90% blood-feeding inhibition in the absence of a physical barrier. A long-term residual efficacy of 12 months was observed by WHO-bioassays in huts treated with two layers (60-80%). Mortality after an overnight exposition at distances of 1 meter was 96-100% for up to 12 months.

**Conclusion:**

The encouraging results obtained on the insecticide paint Inesfly 5A IGR™ in terms of mortality, be it in direct contact or at a distance, and its new operational approach could constitute an additional option in malaria control efforts in areas of pyrethroid resistance. Phase III studies will be performed to assess the product's epidemiological impact and sociological acceptance.

## Background

Primary prevention of malaria on a large scale is essentially achieved through vector control. Currently, the two main vector control methods: 1) indoor residual insecticide spraying (IRS), and 2) insecticide-treated nets (ITNs), aim at the primary protection of individuals and populations against the bite of infected *Anopheles *mosquitoes [1,2]. Pyrethroids are presently the only insecticides recommended for treatment of mosquito nets because of their rapid knockdown, high insecticidal potency at low dosages, and relative safety for mammals [3]. While both, IRS and ITNs have been found to be efficient and cost-effective across a large number of settings [1] it is not clear whether these interventions alone will achieve those critical low levels of transmission that result in successful malaria vector control. Moreover, because of i) the expanding resistance of main malaria vectors to pyrethroids [4], and ii) operational drawbacks to IRS [5], there is need for novel strategies in the framework of an integrated vector management [6]. Insecticide paint Inesfly 5A IGR™ is a "cocktail" consisting of two organophosphates, chlorpyriphos and diazinon and an insect growth regulator (IGR), pyriproxyfen. The same paint has been evaluated under experimental conditions against *Triatoma infestans*, a main vector of Chagas disease in Argentina [7] and Bolivia [8]. Results showed high mortalities and long residual activity in both cases. The paint was well accepted and tolerated by populations exposed to it [8]. Studies performed at the Instituto de Salud Carlos III in Spain have shown the paint's safety in terms of irritancy (ocular, dermal and systemic), cytotoxicity and mutagenicity [9].

The efficacy and residual effect of Inesfly 5A IGR™ insecticide paint has been tested in the laboratory at LIN (Laboratoire de Lutte contre les Insectes Nuisibles) of the Institut de Recherche pour le Développement (IRD) in Montpellier, France, on different kinds of surfaces using laboratory strains of 100% OP-resistant and 100% OP-susceptible *Culex quinquefasciatus*. A residual efficacy of over 12 months was observed on most surfaces even against resistant mosquitoes (Mosqueira *et al.*, submitted). Community adherence to malaria control measures is higher if strategies are also effective against nuisance [10-12] which may be further complicated since the pest mosquito *Cx. quinquefasciatus *has become resistant to the most common insecticides used for bed net impregnation [13].

The objective of the present study was to evaluate the entomological efficacy and the residual effect of Inesfly 5A IGR™ insecticide paint in experimental huts in Benin, West Africa, against local wild pyrethroid-resistant populations of the major malaria vector, *Anopheles gambiae*, and pest mosquito, *Cx. quinquefasciatus*, for one year.

## Methods

### Study site

Ladji (6◦23N-2◦25) is a large village located by the Nokoué Lake that floods during the rainy season creating breeding sites for *An. gambiae*. The local population of *An. gambiae *is comprised entirely of the M molecular form and shows resistance to pyrethroids and DDT, *kdr *is present at a high frequency, but is susceptible to organophosphates and carbamates, the *ace-1*^*R *^mutation was absent [14]. Pest mosquito *Cx. quinquefasciatus *is also present all year round and shows high resistance to DDT, pyrethroids and carbosulfan with high kdr frequency and elevated levels of esterases and GST activity [14]. The *ace-1*^*R *^mutation was absent [14].

### Insecticide paint

Inesfly 5A IGR™ contains two organophosphates, chlorpyriphos (1.5%) and diazinon (1.5%) and an insect growth regulator (IGR), pyriproxyfen (0.063%), as active ingredients. The formulation is vinyl paint with an aqueous base, with the active ingredients residing within Ca CO3 and resin microcapsules, allowing a gradual release of active ingredients. Microcapsules range from one to several hundred micrometers in size. The paint was applied with a regular brush.

### Early morning collection (EMC)

Inesfly 5A IGR™ was evaluated in 6 experimental huts for over 12 months from September 2003 to September 2004 at the Ladji station. Mosquito collections were performed following WHO testing procedures [15]. Experimental huts were built similarly to those used in Cote d'Ivoire by Darriet *et al *[16]. Huts were treated with one or two layers of insecticide paint at 1 kg commercial product/6 m^2^. Huts treated with two layers had the first layer diluted in 20% water following manufacturer's recommendations. The overall random disposition of huts was: H1: Control 1 (no paint); H2: one layer of insecticide paint on walls; H3: one layer of insecticide paint on walls and ceiling; H4: two layers of insecticide paint on walls; H5: Control 2 (Inesfly paint with no insecticide); and H6: two layers of insecticide paint on walls and ceiling. Team members working in mosquito collection were informed in writing and orally (though they were all literate) about the study and were given the time to think before giving Informed Consent. All team members were provided with intact non-treated bed nets to protect them. Ethical authorization for this research was obtained from the Ministry of Health. Confirmed *Plasmodium falciparum *parasitaemia would be treated as per Benin's Ministry of Health's recommendations. Before treating, mosquitoes were collected for several nights to check that there was no difference between huts in attractiveness to mosquitoes. Though generally done, in this study it was even more important since treatments could not be rotated. To reduce the effect of variation in individual attractiveness to mosquitoes, sleepers rotated between huts on successive study nights. Mosquito collections were performed for thirteen weeks during the first three months; and for six weeks minus/plus three weeks on time points 6, 9 and 12 months after treatment. Following WHO Phase II procedures, four entomological criteria were evaluated: (i) deterrent effect, (ii) excito-repellent effect, (iii) blood feeding inhibition, (iv) mortality rate.

Operationally speaking, the greatest advantage was found if and when females have not had an opportunity to blood-feed before they die. Blood-feeding inhibition rates leave the question open as to whether females would blood-feed the next day on some other individual. The product's impact on blood-feeding has been interpreted in terms of unfed mortality in treated *vs *control huts.

### Mosquito release experiments

On two occasions, mosquito bed nets were removed to assess blood-feeding in the absence of a physical barrier. Mosquitoes used were malaria-free five-day old unfed *An. gambiae *females bred at CREC's insectarium from wild larvae caught at Ladji. Females were released in batches of 100 females per hut at 21:00, just after volunteers entered huts. The next morning, females were collected as per Early Morning Collections. Two replicates were performed at the start of the evaluation (T0).

### Residual efficacy tests

Thirty-minute standard WHO cone bioassays [17] were carried out using 3-5 day old unfed females of *Cx. quinquefasciatus *S-Lab and *An. gambiae *Kisumu, both reference strains susceptible to all insecticides reared at the CREC insectarium. Tests were performed every three months after treatment.

### Distance tests

Unfed females of *An. gambiae *Kisumu and *Cx. quinquefasciatus *S-Lab, 3-5 day old, reared at the CREC insectarium, and susceptible to all insecticides, were introduced into four 150-ml cups, with 15 females per cup per hut. Mosquito netting was placed at both ends to allow air to go through. Honey-soaked cotton was introduced to ensure that females did not die from starvation. Tubes containing females were placed horizontally inside huts from 19:00 to 7:00 h, at a distance of 1 m from two perpendicular walls. The following morning, females were taken to the insectarium for mortality assessment after 24 hours at 80 ± 10% relative humidity and 27 ± 2°C temperature. Tests were performed every three months after treatment.

### Statistical analysis

χ^2 ^analyses were run to test whether differences were statistically significant. EMC and Mosquito release experiments: The Statcalc application of Epi-Info 6 (USD, Inc., Snellville, U.S.A.) was used to analyse differences in exophily, blood-feeding and mortality rates among huts; to analyse differences in entry rates, ANOVA was used. When mortality rates in control huts were between 5 and 20% Abbott's mortality correction formula was applied. Residual efficacy and distance tests: Immediate and delayed mortality were analysed using Epi-Info 6. Where values were <5, Fisher exact tests were used. Because bioassay tests are subject to variations, a 99% confidence interval was applied.

## Results

### Early morning collection (EMC)

As is common for OPs, no deterrent or excito-repellent effect was observed neither against *An. gambiae *nor *Cx. quinquefasciatus*. For the first three months, 100% of *An. gambiae *females in huts treated with two layers, and 76% in huts treated with one layer, died before blood feeding (Table 1) while only 12% died without blood feeding, in control huts. In the case of *Cx. quinquefasciatus*, 88% of females died unfed in huts with two layers and 80% in huts with one layer, while only about 3% died unfed in control huts (Table 2). Nine months after treatment, 83% of *An. gambiae *died unfed in huts treated with two layers on walls, and 59% on huts treated with two layers on walls and ceiling - this difference is due to the fact that the bed net was not fixed correctly in the hut treated with two layers on walls and ceiling for a week during the short period when we had most *An. gambiae *coming in. On huts treated with one layer on walls, 33% of *An. gambiae *died unfed (the only rate not significantly different from control), while a rate of 72% was observed in huts treated with one layer on walls and ceiling. Mortality of unfed females in control huts was 12-14%. In the case of *Cx. quinquefasciatus*, 6% of females died unfed in control huts, while 51-54% died unfed in both huts treated with two layers. On huts treated with one layer on walls, 22% of *Cx. quinquefasciatus *died unfed and 40% in huts treated with one layer on walls and ceiling. By 12 months after treatment, mortality rates of unfed females fell to near control levels for both species.

**Table 1:** Overall mortality and unfed mortality of Anopheles gambiae females collected from experimental huts during EMCs.

EMC *Anopheles gambiae*		Untreated bed net (Control)	Untreated bed net + 2 layers Control Paint on walls and ceiling	Untreated bed net +1 layer IP on walls	Untreated bed net +1 layer IP on walls and ceiling	Untreated bed net +2 layers IP on walls	Untreated bed net +2 layers IP on walls and ceiling
T0-T3	% Overall Mortality	0^a^	0^a^	100^b^	100^b^	100^b^	100^b^
	% Unfed Mortality	12.5^a^	11.1^a^	75^b^	77.8^b^	100^c^	100^c^

T9	% Overall Mortality	0^a^	0.9^a^	34.6^a^	79.7^b^	90.2^b^	93.1^b^
	% Unfed Mortality	15^a^	14^a^	33.3^a,c^	72.4^b^	83.3^b^	58.8^c^

IP = Insecticide Paint. T0-T3 and T9 = 0-3 and 9 months after treatment

Values in the same row sharing a letter superscript do not differ significantly (P ≥ 0.05)

**Table 2:** Overall mortality and unfed mortality of Culex quinquefasciatus females collected from experimental huts during EMCs.

EMC *Culex quinquefasciatus*		Untreated bed net (Control)	Untreated bed net + 2 layers Control Paint on walls and ceiling	Untreated bed net +1 layer IP on walls	Untreated bed net +1 layer IP on walls and ceiling	Untreated bed net +2 layers IP on walls	Untreated bed net +2 layers IP on walls and ceiling
T0-T3	% Overall Mortality	0^a^	0^a^	100^b^	100^b^	100^b^	100^b^
	% Unfed Mortality	3.4^a^	2.1^a^	81.2^b^	79.4^b^	87.8^c^	88^c^

T6	% Overall Mortality	0^a^	2.2^a^	92.9^b^	95.7^c^	100^d^	99.5^d^
	% Unfed Mortality	5.6^a^	7.6^a^	78.3^b^	70.1^b,c^	69.4^b,c^	84.5^b,d^

T9	% Overall Mortality	0^a^	2.1^a^	20.8^b^	40.1^c^	56.7^d^	54.5^d^
	% Unfed Mortality	2.7^a^	4.3^a^	22^b^	39.5^c^	53.7^d^	50.7^d^

T12	% Overall Mortality	0^a^	1.2^a^	5.7^b^	5.3^b^	15.6^c^	21.6^d^
	% Unfed Mortality	5^a^	7^a,b^	9.7^b^	7.9^a,b^	17^c^	23.9^d^

IP = Insecticide Paint. T0-T3, T6, T9 and T12 = 0-3, 6, 9 and 12 months after treatment

Values in the same row sharing a letter superscript do not differ significantly (P ≥ 0.05)

Mortality was 100% up to three months against both, local populations of *An. gambiae *and *Cx. quinquefasciatus *for all treated huts, differences being significant compared to control. Six months after treatment, mortality rates against *Cx. quinquefasciatus *were of 90-100% for all treated huts (Table 2). Due to seasonal factors, there is no data on *An. gambiae *for that time point. By nine months after treatment, mortality rates in huts treated with two layers were still 90-93% against *An. gambiae *and 54-57% against *Cx. quinquefasciatus *(Tables 1 and 2, respectively). By twelve months, mortality was still higher compared to control in huts treated with two layers (p < 10^-3^) and one layer (p < 0.05) (Table 2).

### Mosquito release experiments

**Figure 1:**
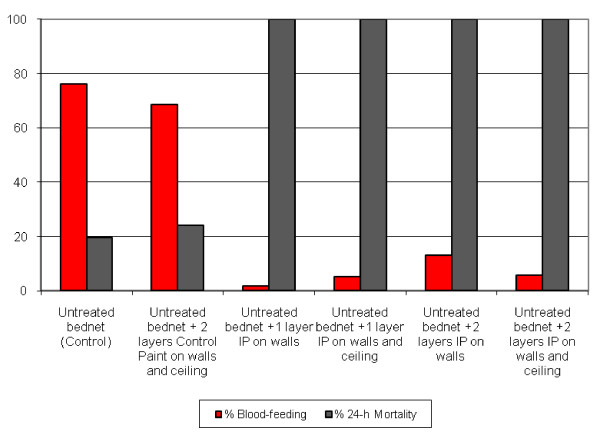
**Delayed 24-hour mortality and blood-feeding rates during mosquito release experiments of local *Anopheles gambiae***. Malaria-free females reared at the CREC insectarium were released into each hut at 21:00 hours and collected between 5 and 7 hours the next day. Bed nets had been withdrawn and mosquito entry into the huts was blocked. Averages from two repeats of N > 30 each.

Blood-feeding in treated huts went from 2 to 13%, whereas control huts yielded blood-feeding rates of 68.5 and 76.1% (Figure 1). Differences between treated and control huts were significantly different (p < 10^-3^).

### Residual efficacy tests

In huts treated with one layer, mortality rates of 98-100% were observed against both *An. gambiae *Kisumu and *Cx. quinquefasciatus *S-Lab for up to three months (Tables 3 and 4). *Anopheles gambiae*, mortality rates started dropping six months after treatment to values of 79.4 and 59.7%. *Culex quinquefasciatus *values of 98-100% continued to be observed 6 and 9 months after treatment. At nine months after treatment, mortality rates dropped to 14.7% against *An. gambiae *(Table 3). In huts treated with two layers, mortality rates of 98-100% were observed for both *An. gambiae *and *Cx. quinquefasciatus *for up to nine months (Tables 3 and 4). Twelve months after treatment mortality rates were of 70-80% against *An. gambiae *and *Cx. quinquefasciatus*.

**Table 3:** Delayed 24-hour mortality of Anopheles gambiae Kisumu after a 30-minute exposure to treated and control walls.

WHO Bioassays% Mortality *Anopheles gambiae *Kisumu	Untreated bed net (Control)	Untreated bed net + 2 layers Control Paint on walls and ceiling	Untreated bed net +1 layer IP on walls	Untreated bed net +1 layer IP on walls and ceiling	Untreated bed net +2 layers IP on walls	Untreated bed net +2 layers IP on walls and ceiling
T0	12.5^a^	14.1^a^	100^b^	100^b^	100^b^	100^b^

T3	0^a^	3.3^a^	100^b^	100^b^	100^b^	100^b^

T6	0^a^	1.8^a^	79.4^b^	59.7^c^	100^d^	100^d^

T9	0^a^	3.4^a, b^	14.7^b^	44.6^c^	100^d^	98.5^d^

T12	1.7^a^	6.1^a, b^	0^a^	12.9^b^	80.6^c^	71.9^c^

IP = Insecticide Paint. T0, T3, T6, T9 and T12 = 0, 3, 6, 9 and 12 months after treatment

Values in the same row sharing a letter superscript do not differ significantly (P ≥ 0.05)

**Table 4:** Delayed 24-hour mortality of Culex quinquefasciatus S-Lab after a 30-minute exposure to treated and control walls.

WHO Bioassays% Mortality *Culex quinquefasciatus *S-lab	Untreated bed net (Control)	Untreated bed net + 2 layers Control Paint on walls and ceiling	Untreated bed net +1 layer IP on walls	Untreated bed net +1 layer IP on walls and ceiling	Untreated bed net +2 layers IP on walls	Untreated bed net +2 layers IP on walls and ceiling
T3	5.5^a^	6.2^a^	100^b^	100^b^	100^b^	100^b^

T6	13.8^a^	10.3^a^	100^b^	98.3^b^	100^b^	100^b^

T9	1.6^a^	3.3^a^	72.6^b^	49.2^c^	100^d^	98.4^d^

T12	1.6^a^	0^a^	5^a^	8.1^a^	70^b^	72.4^b^

IP = Insecticide Paint. T0, T3, T6, T9 and T12 = 0, 3, 6, 9 and 12 months after treatment

Values in the same row sharing a letter superscript do not differ significantly (P ≥ 0.05)

### Distance tests

Huts treated with one layer yielded mortalities of 90-100% against *An. gambiae *Kisumu (Table 5) and *Cx. quinquefasciatus *S-Lab (Table 6) for up to six months. By 12 months, a volume effect was observed in the hut treated with one layer just on walls (35.6% for *An. gambiae *and 60% *Cx. quinquefasciatus*) versus that treated on walls and ceiling (98.4% for *An. gambiae *and 96.2% *Cx. quinquefasciatus*), but differences were still significant with respect to control (p < 10^^-6^^) for both. Huts treated with two layers yielded mortalities 100% against *An. gambiae *and *Cx. quinquefasciatus *for 12 entire months (Tables 5 and 6).

**Table 5:** Delayed 24-hour mortality of Anopheles gambiae Kisumu after an overnight exposure at a distance of one meter from two perpendicular walls.

Distance tests% Mortality *An. gambiae *Kisumu	Untreated bed net (Control)	Untreated bed net + 2 layers Control Paint on walls and ceiling	Untreated bed net +1 layer IP on walls	Untreated bed net +1 layer IP on walls and ceiling	Untreated bed net +2 layers IP on walls	Untreated bed net +2 layers IP on walls and ceiling
T0	0^a^	3.4^a^	100^b^	100^b^	100^b^	100^b^

T6	0^a^	0^a^	91.8^b^	100^b^	100^b^	100^b^

T12	1.5^a^	3^a^	35.6^b^	98.4^c^	100^c^	100^c^

IP = Insecticide Paint. T0, T6 and T12 = 0, 6 and 12 months after treatment

Values in the same row sharing a letter superscript do not differ significantly (P ≥ 0.05)

**Table 6:** Delayed 24-hour mortality of Culex quinquefasciatus S-Lab after an overnight exposure at a distance of one meter from two perpendicular walls.

Distance tests% Mortality *Culex quinquefasciatus *S-Lab	Untreated bed net (Control)	Untreated bed net + 2 layers Control Paint on walls and ceiling	Untreated bed net +1 layer IP on walls	Untreated bed net +1 layer IP on walls and ceiling	Untreated bed net +2 layers IP on walls	Untreated bed net +2 layers IP on walls and ceiling
T0	8.3^a^	0^a^	100^b^	100^b^	100^b^	100^b^

T6	13.8^a^	10.3^a^	100^b^	98.3^b^	100^b^	100^b^

T12	1.8^a^	3^a^	60^b^	96.2^c^	100^c^	100^c^

IP = Insecticide Paint. T0, T6 and T12 = 0, 6 and 12 months after treatment

Values in the same row sharing a letter superscript do not differ significantly (P ≥ 0.05)

## Discussion

The efficacy of Inesfly 5A IGR™ was tested against pyrethroid-resistant *An. gambiae *and *Cx. quinquefasciatus*. Contrary to the results obtained by N'Guessan *et al *[18] and Assidi *et al *[19] in experimental huts, when testing OPs, neither a deterrent nor an exito-repellent effect was observed throughout the trial.

The product's best profile was found to be its capacity to kill mosquitoes. Mortality rates as high as 100% were obtained up to three months against both species. A nine-month residual efficacy was observed through bioassay testing as well as through Early Mosquito Collection, analogous to the nine-month residual activity obtained with chlorpyrifos-methyl applied by IRS in the same study area against the same mosquito populations of Ladji in Cotonou [18]. Mosquito killing was quick enough to prevent blood feeding: during mosquito release experiments, in the absence of the physical barrier provided by bed nets, only 2 to 13% of females blood fed in treated huts, whereas blood feeding in control huts was 72%, similar to the 83% obtained by Darriet *et al *[16] in Ivory Coast in huts with no bed nets. These findings were supported by Early Morning Collection data, where the number of females that died in treated huts without having blood-fed was significantly different compared to control.

Mortality rates observed in distance experiments were most striking. Females placed overnight at distances of one metre from treated walls died even twelve months after treatment. Because even highly endophilic pest or vector mosquitoes are not always in contact with an insecticide-treated surface before contacting a human or animal host, especially on pyrethroid-treated surfaces due to its excito-repellent effect, it is desirable to have a distance effect. The lethal effect at a distance observed in the insecticide paint goes in this direction. A possible mass protective effect as a result of mass house-treatment needs to be studied. On a product safety note, Acute Inhalation Toxicity studies classified this paint as Category III (according to WHO) and category IV (according to EPA) - no warning label required in either case [20].

As results show, a "layer effect"and a "volume effect" was observed by all three tests, EMC experiments, bioassays and distance tests. The "layer and volume effect" became more evident with time. Porous surfaces like cement benefited from treatment with two layers. Similarly, huts treated with only one layer benefited particularly from the treatment of a larger volume. Whether subsequent layers prolong the product's long lasting efficacy needs to be explored.

To test whether efficacy hinged more on porosity than dose, a parallel study was performed. Cement-made surfaces painted with a control layer and an insecticide paint layer at 1 kg/6 m^2^, performed as well as two insecticide paint layers at 1 kg/6 m^2^, even though the latter had twice the dose (Mosqueira *et al.*, unpublished data).

The paint offers a different operational approach that could be of value. Unlike IRS, people are able to apply the paint themselves, no need of trained personnel or special equipment. Homes' appearance would also improve leading, potentially, to changes in behaviour of public health significance [21].

Findings suggest the potential value of the insecticide paint as a vector control tool in areas of pyrethroid resistance and in urban settings. While it is clear that urban malaria represents a major challenge for public health in Africa [22], several factors make urban environments suitable for the insecticide paint: 1) superior resources, 2) the paint's effectiveness against nuisance; 3) population densities would facilitate coverage and a potential mass effect; 4) the vast majority of houses and public spaces, such as hospitals, schools, prisons, churches and mosques, are made of surfaces suitable for painting.

## Conclusions

The lethal effect of the insecticide paint observed in the field against local populations of *An. gambiae *and *Cx. quinquefasciatus *resistant to pyrethroids was encouraging. Killing was not only high but quick enough to prevent blood feeding. A residual efficacy of nine months was observed as per mosquito collections and 30-minute bioassays. Females left overnight at distances of one meter continued dying significantly even after 12 months. The possible existence of a mass-effect needs to be studied in a large-scale epidemiological setting. Future endeavours will be directed towards the study of the insecticide paint's efficacy on the incidence of malaria as well as its acceptability.

## Competing interests

The authors declare that they have no competing interests.

## Authors' contributions

PC and SMC conceived the protocol. PC, SMC, FC and BM contributed to the design of the study. FC and JMH critically contributed to the implementation of the study. JC and BM conducted evaluations. MA was the director of the Centre de Recherche Entomologique de Cotonou (CREC). The manuscript has been drafted by BM and has been revised by PC. All authors read and approved the final manuscript.
